# The social determinants of migrant domestic worker (MDW) health and well-being in the Western Pacific Region: A Scoping Review

**DOI:** 10.1371/journal.pgph.0002628

**Published:** 2024-03-27

**Authors:** Jamie Chan, Georgia Dominguez, Antonia Hua, Melissa Garabiles, Carl A. Latkin, Brian J. Hall

**Affiliations:** 1 Department of International Health, Johns Hopkins University Bloomberg School of Public Health, Baltimore, Maryland, United States of America; 2 Centre for Global Health Equity, NYU Shanghai, Shanghai, People’s Republic of China; 3 Centre for Global Child Health, The Hospital for Sick Children, Toronto, Canada; 4 Psychology Department, De La Salle University, Manila, Philippines; 5 Department of Health, Behavior and Society, Johns Hopkins University Bloomberg School of Public Health, Baltimore, Maryland, United States of America; Bielefeld University, GERMANY

## Abstract

The health and well-being of transnational migrant domestic workers (MDWs) is a pressing but largely neglected public health concern. The Asia Pacific region is home to over 20% of the global MDW population. Living and working conditions, social contexts, political environments, and migration regimes are recognized as consequential to the health of this population, but currently no synthesis of available literature to prioritize research or policy agenda setting for MDW has yet been conducted. This scoping review screened 6,006 peer-reviewed articles and 1,217 gray literature sources, identifying 173 articles and 276 gray literature sources that reported key MDW health outcomes, social determinants of health, and related interventions. The majority of identified studies were observational and focused on the prevalence of common mental disorders and chronic physical conditions, with most studies lacking population representativeness. Identified social determinants of health were primarily concerned with personal social and financial resources, and health knowledge and behaviors, poor living and working conditions, community resources, experienced stigma and discrimination, poor healthcare access, exploitation within the MDW employment industry, and weak governance. Six interventional studies were identified that targeted individual-level health determinants such as financial and health knowledge with mixed effectiveness. Future population representative epidemiological and respondent driven sampling studies are needed to estimate population health burdens. In addition, randomized control trials and public health intervention studies are needed to improve women’s health outcomes and address proximal health determinants to reduce health inequalities. Leveraging social networks and community facing non-governmental organizations (NGOs) are promising directions to overcome access to care for this population.

## Introduction

### Rationale

The number of transnational migrant workers in the Asia Pacific has increased steadily since the late 20^th^ century, from 52 million in 1990 to 65 million in 2019 (25% of international migrants globally in 2019) [1], with over two-thirds of all migration occurring intra-regionally (e.g., from the Philippines to China) [1].

Rates of intra-regional migration for opportunities in domestic and care sectors have surged in the Western Pacific in the 21^st^ century [2, 3], and this number is projected to rise, given the increasing care needs of ageing populations, rates of women entering the workforce, and continued gendered division of domestic and care work in the region [3–5]. Countries and territories in the Western Pacific host some of the greatest share of migrant domestic workers (MDWs) in the world. According to the ILO, Asia Pacific countries host over 20% of the world’s 11.5 million MDWs [2]. In Hong Kong, MDWs constitute almost 10% of the total working population, while the proportion in Macau and Singapore is approximately 7% (see Table 1) [6–11].

**Table 1:** Total MDW population and country of origin by host country or territory.

Host Country or Territory, Year	Total MDW Population	MDW Country of Origin
Hong Kong, 2021 [9]	339,451	Philippines: 191,783 (56.5%)
		Indonesia: 140,057 (41.3%)
		Others: 7,611 (2.24%)
Singapore, 2022 [7]	256,300	Data unavailable
Taiwan, 2022 [12]	231,572	Indonesia: 184,694 (79.8%)
		Philippines: 27,173 (11.7%)
		Vietnam: 19,330 (8.3%)
		Thailand: 374 (0.2%)
		Other: 1
Malaysia, 2022 [13]	86,084	Indonesia: 59,605 (69.2%)
		Philippines: 22,803 (26.5%)
		Vietnam: 1,031 (1.2%)
		Cambodia: 976 (1.1%)
		Others: 1,669 (1.9%)
Macau, 2022 [10]	23,135	Philippines: 13,030 (56.3%)
		Vietnam: 4,534 (19.6%)
		Indonesia: 3,202 (13.8%)
		Myanmar: 1,159 (5.0%)
		Mainland China: 1,015 (4.4%)
		India: 56 (0.2%)
		Thailand: 28 (0.1%)
		Taiwan Region: 16 (0.07%)
		Others: 71 (0.3%)

*Note*. MDW = Migrant domestic worker

MDWs are transnational migrants who are formally employed to carry out domestic work in households [14–16]. Globally, over 80% of MDWs are women [2]. Hired to perform essential household tasks including cooking and cleaning—but with an expanded scope of work to include child and elder care—they play significant roles in local labor markets, remittance economies, and increasingly complex and expanding transnational care chains [17]. While sending countries benefit from large remittance inflows, receiving countries benefit significantly from the employment of low-wage domestic labor, allowing women in receiving countries to take up jobs outside the household [18, 19].

Nearly all MDWs in the top destination locations in the Western Pacific region are women and of child-bearing age [20–27]. The primary underlying drivers of migration for MDWs are economic. With large disparities between local and overseas wages and high levels of local unemployment and underemployment, women migrate in search of opportunities to advance their families’ livelihoods and socioeconomic status [28–30].

In the past decade, there has been an increase in research on migrant health, specifically on MDW health outcomes and barriers to health [31–36]. This work highlights the multitude of ways MDWs face social and health inequities in various domains—ranging from the workplace and healthcare settings to policy and migration industry [37, 38]. A United Nations (UN) report described how women migrant workers are at greater risk of occupational violence and abuse compared to male migrant workers [39]. In 2010, only 10% of domestic workers around the world were covered by general labor laws to the same extent as non-domestic workers [40]. In the Asia Pacific, the majority (61%) were excluded entirely from general labor laws [40]. More recently, research conducted during the COVID-19 pandemic highlighted systemic health inequities and barriers to healthcare and safety confronted by MDWs, ranging from increasingly restrictive working conditions to a lack of access to health protection resources [41–48].

Given the recent growth in research on MDW health in the Western Pacific Region, there is a need for a synthesis of the literature to consolidate key findings, highlight knowledge gaps, and identify areas of future research and intervention development. While previous scoping reviews explored the academic literature on MDW health, they did not have a specific geographical focus and primarily examined MDW health outcomes or proximal health stressors, such as workplace abuse, help-seeking, and social support [49–51]. Previous scoping reviews focused primarily on the health outcomes and experiences of MDWs while they were working in the host country. Considering increasing emphasis of pre- and post-migration health determinants in migration research, there is a gap in understanding of health determinants for MDWs across the migration journey [52, 53]. Additionally, only one scoping review article, focused on MDW peer support, included literature after 2019 [54]. Therefore, the existing literature largely excludes more recent developments that may be linked to MDW health, including studies conducted during the COVID-19 pandemic.

The aim of this scoping review is to fill a crucial gap in the literature on MDW health, synthesizing the available peer-reviewed and gray literature identified through our search strategy on MDW health and well-being in the Western Pacific Region. It shifts the emphasis from health outcomes to health determinants and interventions, in parallel with efforts of the International Organization for Migration (IOM), the International Labour Organization (ILO), and the United Nations (UN) Global Compact for Safe, Orderly, and Regular Migration [55–59].

### Objectives

The scoping review addresses the following research questions:

1. What are the most prevalent physical and mental health conditions, illnesses, and needs of MDWs in the Western Pacific Region?
2. What are the key social determinants of poor physical and mental health among MDWs in the Western Pacific Region?
3. What is the evidence of effective interventions to improve MDW health outcomes or the determinants of MDW health in the Western Pacific Region?
4. What are the gaps in the existing literature on MDW health in the Western Pacific Region?

## Methods

### Protocol and registration

The protocol was developed according to the PRISMA extension for scoping reviews (PRISMA-ScR). The protocol was finalized and registered online on Open Science Framework on 19 August 2022 (https://osf.io/4tnqp/).

### Eligibility criteria

Consistent with the ILO, ‘migrant domestic workers’ were defined as individuals who migrate to another country for formal employment and to perform full-time domestic work in a household [60]. Live-in domestic workers who reside in their employers’ household and live-out workers who have separate accommodation outside of the workplace were included. We also included migrants with different lengths of stay and migration histories. For example, cyclical or circular migration involves temporary returns home between short-term contracts abroad based on individual or family needs [28, 61]. Serial migration or stepwise migration involves sequential contracts in different countries with the goal of increased mobility, securing better working conditions and salaries, or permanent residence in onward destinations [62–64].

‘Social determinants of health and well-being’ were defined in alignment with the WHO as any non-medical factors, circumstances, and conditions that influence health and well-being outcomes [65]. We used the migrant health determinants identified in the IOM’s adaptation of the WHO social determinants of health framework to develop our health determinants data categories in this scoping review: individual factors; lifestyle factors, living conditions; working conditions; social and community factors; and governance and socioeconomic conditions [56].

Peer-reviewed studies conducted in any of the 37 countries, territories, and areas located in the WHO Western Pacific Region were eligible for inclusion. Only articles published in English after 1 January 2000 were included. The scoping review excluded peer-reviewed studies 1) that focused either on migrant workers not explicitly employed to perform domestic work, as well as domestic workers who are working in their country of origin; 2) conducted outside of the Western Pacific region; 3) with interventions that were not specifically aimed at MDW health outcomes or determinants; 4) with outcomes other than MDW health, well-being, health determinants or interventions addressing MDW health or determinants; and 5) published as conference abstracts, dissertations, theses, books and book chapters, book or film reviews, letters to the editor, editorials, historical narratives, case studies, opinion articles, or commentaries.

For the gray literature search, sources included those from locations that either send or receive sizeable populations of MDWs, as this provided a more holistic understanding of the policies and circumstances shaping workers’ health and well-being. An initial gray literature search encompassing only receiving countries found limited and skewed literature on MDW health determinants and interventions, likely because the onus has historically been placed on sending countries to protect migrant health. The review included any gray literature sources that discussed health outcomes and determinants of MDWs who had worked or were presently working in the Western Pacific, and any interventions that affected the health and well-being of MDWs who had worked or were presently working in the Western Pacific.

### Search and selection

EMBASE, PsycINFO, PubMed, and Web of Science databases were searched electronically for relevant peer-reviewed literature published between 1 January 2000 and 30 September 2023 (S1 Appendix). 7,517 peer-reviewed articles were identified through the initial search. We used backward snowballing methods, looking at the reference lists of included peer-reviewed articles to identify additional records for inclusion. Duplicate peer-reviewed articles were removed using Covidence, and titles and abstracts were screened according to the inclusion and exclusion criteria, with 173 peer-reviewed articles included for data extraction. The peer-reviewed articles were screened by one author (JC) with consultation by an additional reviewer (BJH). For the gray literature, two authors (HA, GD) conducted single screening, with 10% of screened articles cross-checked by a third author (JC) and discrepancies resolved through discussion with the team.

The gray literature sources are displayed in S3 Appendix. 1,202 gray literature records were identified through the initial search. Gray literature sources were assessed based on inclusion criteria, with 276 gray literature records included in the review.

### Data items, charting, and synthesis

Data extraction forms for the peer-reviewed and gray literature were developed in Excel based on the scoping review research questions and frameworks (S2 Appendix). All peer-reviewed data were extracted by one independent reviewer (JC), and uncertainties were discussed in the study team. The grey literature data were extracted by two reviewers (AH, GD) and cross-checked by a third reviewer (JC). The descriptive summaries across the peer-reviewed and grey literature were combined in a table to conduct thematic analyses of health outcomes, health determinants, and interventions.

The WHO framework on health determinants adapted by the IOM [56], as well as the IOM and Zimmerman et al. frameworks on migration phases [53], were used to categorize the health determinants data.

An increasing number of studies focus on how diverse socioeconomic, political and cultural factors shape the health of migrants, and particularly for those in domestic work [35, 66–72]. The IOM’s adaptation of the WHO social determinants of health framework explores how diverse factors at all levels of the social ecology affect the health and well-being of migrants, identifying the following overarching domains, from most proximal to most distal: individual factors; lifestyle factors; living conditions; working conditions; social and community factors; and governance and socioeconomic conditions [56]. After an initial review of the titles and abstracts of the included literature, we decided to adapt and expand the categories to more comprehensively and accurately capture the health determinants evidenced in the data, adding more specific data categories including intrapersonal factors; knowledge and behaviors; community resources; stigma and discrimination; healthcare access; and the migration industry.

The IOM and Zimmerman et al. developed frameworks to show how migrants face different health determinants across various phases of migration—pre-migration; peri-migration (or movement, arrival and integration); and post-migration or return to country of origin [53, 56]. Given that previous scoping reviews focus primarily on the health outcomes and experiences of MDWs in the peri-migration phase, we aimed to identify different MDW health determinants across the migration journey, including pre- and post-migration.

## Results

### Overview

**Fig 1:**
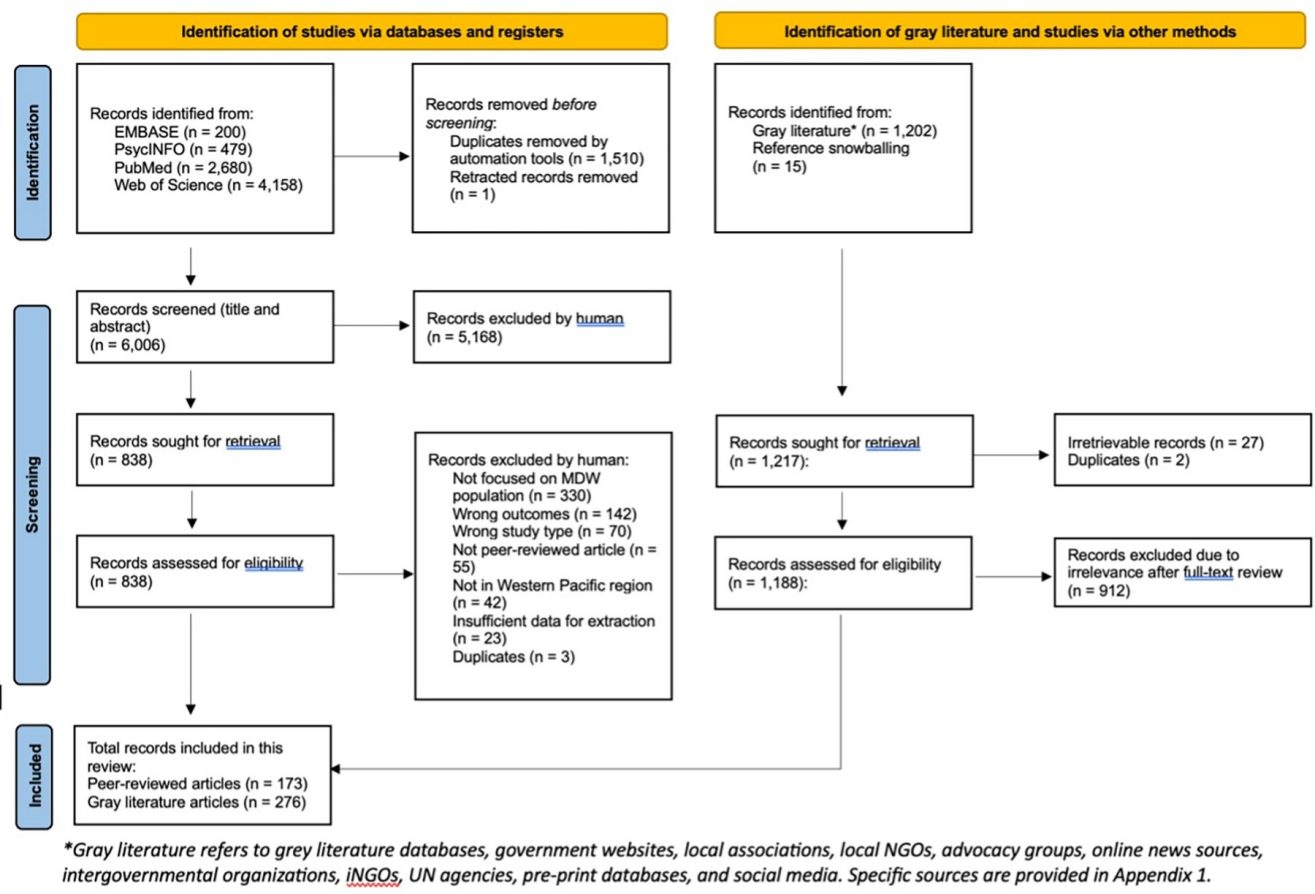
PRISMA flow diagram of scoping review.

A total of 173 peer-reviewed studies and 276 gray literature records were included in the scoping review. The results of the literature search and screening are depicted in Fig 1.

All peer-reviewed studies were conducted in either Hong Kong (n = 85), Singapore (n = 57), Macau (n = 20), Taiwan (n = 10), or Malaysia (n = 8), with one study conducted in mainland China. Several gray literature types were identified (Table 2), with newspaper articles comprising the majority. The gray literature sources were primarily based in Hong Kong (n = 151), Singapore (n = 53), and the Philippines (n = 29). Peer-reviewed studies and gray literature articles were mainly focused on the health and well-being of MDWs from the Philippines and Indonesia, with several records including MDWs from Myanmar, Thailand, Sri Lanka, India, and unspecified countries in South Asia.

**Table 2:** Summary of peer reviewed and gray literature articles locations, study design/article types, and numbers of health outcomes and determinants.

Source type	Location [ASEAN, Asia Pacific = AP, Bangladesh = BGD, Brunei = BN, Cambodia = KH, China = CN, Global = GL, Hong Kong = HK, Indonesia = ID, Macau = MAC, Malaysia = MY, Myanmar = MM, Philippines = PH, Singapore = SG, South Asia = SA, Southeast Asia = SEA, South Korea = KR, Sri Lanka = LK Taiwan = TW, Thailand = TH, Vietnam = VN, Western Pacific Region = WPR]	Study Design/Article Type	Health Outcomes [Physical Health = PH, Mental Health = MH, Not Reported = NR]	Health Determinants [Community Resources = CR, Healthcare Access = HA, Intrapersonal Resources = IR, Knowledge and Behaviours = KB, Living and Working Conditions = LW, Migration Industry = MI, Policies and Governance = PG, Stigma and Discrimination = SD
Peer-reviewed studies (n = 173)	85 HK 57 SG 20 MAC 10 TW 8 MY 5 ID 4 PH 2 MM 1 CN 1 VN	57 Cross-sectional qualitative 45 Ethnography 40 Cross-sectional quantitative 24 Cross-sectional mixed-methods 3 RCT 3 Secondary data analysis 1 Quasi-experimental	14 Both 12 MH 6 PH 141 NR	76 LW 51 CR 39 PG 30 IR 21 KB 19 HA 17 SDF 12 MI 9 NR
Grey Literature (n = 276)	151 HK 53 SG 29 PH 12 ID 11 TW 9 AP 8 ASEAN 8 MY 5 TH 4 MAC 3 MM 2 GL 2 SEA 1 BGD 1 BN 1 CN 1 KH 1 KR 1 LK 1 VN	191 Newspaper article 35 Government website 32 Organisation report/research/brief 9 Document/booklet 6 Organisation press release 3 Organisation website	3 PH 1 MH 1 Both 271 NR	195 PG 117 LW 87 MI 69 CR 46 SD 19 KB 14 HA 13 IR 12 NR

*Note*: Location and health determinants column totals do not add up to total number of peer-reviewed and gray literature articles because some were conducted in multiple locations and discussed multiple health determinants. For the peer-reviewed articles, some multi-location studies were additionally conducted in non-WPR locations.

For more detail of the peer-reviewed literature study designs and study populations, please see S1 Data.

### Health outcomes

A total of 29 peer-reviewed studies and 9 gray literature sources presented health outcomes of MDW populations. The source locations and numbers of records discussing physical health and mental health and well-being outcomes are displayed in Table 3.

**Table 3:** Number of sources (n) reporting health outcomes in each location.

Location\Health Outcomes	Physical Health (n = 21)	Mental Health and Well-Being (n = 25)
Singapore (n = 16)	12	10
Hong Kong (n = 14)	7	8
Macau (n = 9)	5	7
Mainland China (n = 1)	1	1

*Note*. n = total number of studies in each country. Column totals are not reflective of individual country totals given some studies assessed both physical and mental health/wellbeing.

All the included peer-reviewed studies discussed MDWs’ health outcomes while working in the host location. Studies conducted in Singapore were comprised of participants from the Philippines, Indonesia, India, Sri Lanka, Cambodia, and Myanmar. Physical health problems identified among Singapore-based studies included unintentional injuries, back pain, breathing problems, thyroid illness, digestive issues, and HPV [73–78]. Two studies described physical deterioration, pains, and chronic conditions like diabetes among older MDWs from the Philippines, Indonesia, India, and Sri Lanka who had worked overseas for 20 to 30 years [74, 75]. A study on HPV found that 22.2% of participants (n = 226), primarily comprised of Filipino MDWs, tested positive for HPV, although 70% of the positive results were for non-16 and non-18 HPV strains [73]. In terms of mental health and well-being, the main outcomes among samples in Singapore included emotional pain, stress, trauma, and anxiety from caregiving burdens, poor relationships with employers, and separation from children and family [31, 74–76, 78–82]. One study conducted on MDWs (n = 100) engaged in eldercare found that 4% of the study sample met criteria for clinically elevated anxiety [79]. Another study in Singapore found that 73.6% of MDWs (n = 182) reported an overall “good” or “very good” quality of life [31].

The Hong Kong-based studies primarily sampled MDWs from the Philippines and Indonesia. The main physical health outcomes identified in the studies included symptoms of colds and flu-like fever, cough, and throat pain; dental problems; and chronic conditions like asthma and allergies [22, 33, 83, 84]. Three gray literature articles reported MDW occupational injuries and deaths resulting from falls while cleaning apartment windows [85–87]. One study found that MDW participants (n = 1945) scored worse on general self-rated physical and mental health than the general Hong Kong population [22]. Data collected from MDWs (n = 203) in 2020, during the COVID-19 pandemic, found that 70% self-reported their health status in the past month as “good” or “very good,” and over 80% were either “moderately satisfied” or “very satisfied” with their life [88].

Among studies of MDWs in Hong Kong, key mental health and well-being related outcomes included depression, anxiety, sleep problems, psychological distress and stress disorders, loneliness, and worry [32, 83, 84, 89–91]. In samples of Filipino MDWs, one study (n = 346) found that 39% were at risk for high psychological distress [90], while another study (n = 105) reported that 25% were categorized from “mild” to “extremely severe” for symptoms of depression [32]. An NGO report from the gray literature found that among a population of MDWs from the Philippines, Indonesia, Thailand, and Sri Lanka, 34% self-reported being extremely happy (5 on a scale of 1 to 5), while 39% self-reported health problems including fatigue, insomnia, anxiety, and depression [92].

All nine studies conducted in Macau studied health outcomes among Filipino MDWs. 52.9% of one sample (n = 1186) reported “normal” self-reported health status, with 28.8% and 12.2% reporting “good” and “very good” respectively [93]. In terms of physical health, the main outcomes encompassed chronic conditions including hypertension and diabetes; common illnesses such as colds, fevers, skin rashes, and arthritis; and other concerns such as dizziness, pain, fatigue, sleep problems, and overweight [34, 72, 94]. Among 1164 participants who received an HIV/syphilis rapid test, no HIV or syphilis infection was detected [95]. The key mental health and well-being outcomes in the studies of Filipino MDWs in Macau were anxiety, depression, PTSD, stress, burnout, and related symptoms such as chronic fatigue and difficulty concentrating [34, 94, 96–99]. Addictive behaviors involving gambling and alcohol use were also identified in self-report surveys [34]. In one study (n = 1375), 25.8% of participants met criteria for probable anxiety and/or depression [96]; in another sample (n = 131), the prevalence of probable depression and anxiety was 14.5% and 17.6%, respectively [98]. However, a survey of 22 Filipino MDWs in Mainland China also found generally positive self-reported health [35].

### Health determinants

There was a total of 164 peer-reviewed articles and 264 gray literature records presenting data on health determinants across the region. The majority were from Hong Kong and focused on policies and governance (see Table 4).

**Table 4:** Number of peer-reviewed and grey literature sources reporting data on health determinants in each location.

Vietnam (n = 1)*	Sri Lanka (n = 1)*	South Korea (n = 1)*	China (Mainland) (n = 2)	Cambodia (n = 1)*	Brunei (n = 1)*	Bangladesh (n = 1)*	Southeast Asia (n = 4)*	Thailand (n = 3)*	Myanmar (n = 3)*	Macau (n = 5)	Malaysia (n = 13)	Asia Pacific (n = 7)*	ASEAN (n = 8)*	Indonesia (n = 12)*	Taiwan (n = 22)	Philippines (n = 22)*	Singapore (n = 106)	Hong Kong (n = 236)	Location\ Health Determinants
1	1	1	2 [1]	1	1	1	2	3	3	9 [4]	11 [7]	4	8	8	16 [4]	19	53 [13]	113 [11]	Policies and Governance (n = 234)
0	0	0	1 [1]	1	0	0	0	2	2	4 [3]	9 4]	4	3	2	10 [7]	3	56 [31]	107 [29]	Living and Working Conditions (n = 193)
0	0	0	0	0	0	0	0	0	0	4 [4]	0	0	0	1	4 [2]	1	21 [12]	95 [37]	Community Resources (n = 120)
0	1	0	0	0	0	0	1	2	2	2 [2]	3 [2]	3	3	8	2 [1]	3	22 [6]	55 [1]	Migration Industry (n = 99)
0	0	0	0	0	0	0	0	1	0	1 [1]	1	1	0	0	1 [1]	1	10 [5]	50 [11]	Stigma and Discrimination (n = 63)
0	0	0	0	0	0	0	0	0	0	3 [3]	2 [1]	0	0	1	1 [1]	1	16 [12]	21 [14]	Intrapersonal Resources (n = 43)
0	0	0	0	0	0	0	0	0	0	4 [4]	1 [1]	1	0	1	1 [1]	1	11 [3]	21 [12]	Knowledge and Behaviors (n = 40)
0	0	0	1 [1]	0	0	0	0	0	0	4 [4]	2 [2]	0	0	0	1 [1]	1	5 [2]	21 [10]	Healthcare Access (n = 33)

[n] = peer-reviewed studies

* indicates that only gray literature records were identified

Across the included peer-reviewed and gray literature, the majority of health determinants focused on factors during the peri-migration phase (see Table 5). Factors across the three migration phases characterized by Zimmerman et al. were only found for the theme of policies and governance [53]. The analysis also identified evidence of pre-migration determinants relating to community resources, the migration industry, and knowledge and behaviors.

**Table 5:** Evidence provided for different migration phases characterized by Zimmerman et al. for each health determinant in the included peer-reviewed and gray literature [53].

Health Determinant	Pre-migration	Peri-migration	Post-migration
Policies and Governance (n = 234)	X	X	X
Living and Working Conditions (n = 193)		X	
Community Resources (n = 120)	X	X	
Migration Industry (n = 99)	X	X	
Stigma and Discrimination (n = 63)		X	
Intrapersonal Resources (n = 43)		X	
Knowledge and Behaviors (n = 40)	X	X	
Healthcare Access (n = 33)		X	

#### Policies and governance

Among the included records that discussed health determinants, the greatest proportion of articles (n = 234) discussed policies and governance as a determinant of health for MDWs in the Western Pacific. 195 gray literature records and 39 peer-reviewed articles discussed policies and governance. Key policy issues identified by the peer-reviewed and gray literature sources primarily focused on peri-migration factors, including live-in policies—requiring MDWs to live in their place of work—in Hong Kong, Singapore, Malaysia, and Taiwan; the two-week rule in Hong Kong, which requires MDWs to return to their home country if they are unable to secure new domestic employment within two weeks of the end of a contract; exclusion of MDWs from local minimum wage policies; and relative protections and targeted programming or resources for MDWs by the Hong Kong, Macau, Philippines, and Singapore governments.

Only sources in Hong Kong and Taiwan described the existence of a minimum wage for MDWs. However, some study participants still reported being paid below the minimum wage in Hong Kong [22, 63, 100]. This is comparable to gray literature findings from Taiwan, where the minimum wage for MDWs had remained unchanged for 18 years prior to 2015, but is still presently below the minimum wage of the general workforce [101]. Studies discussed the Singaporean Ministry of Manpower’s lack of policy on minimum wages for MDWs and stipulations that MDWs could forego their rest-days in exchange for compensation from their employers [102–105]. Alternatively, gray literature evidence from Taiwan identified an explicit government hotline available for MDWs to seek counsel or file complaints against employers who violate monthly wage and holiday pay regulations [106].

In both peer-reviewed and gray literature based in Malaysia, barriers to healthcare, public services, and workplace protections were linked to their exclusion from Malaysian labor laws, foreign workers’ insurance (SPIPKA), and social security programs (SOCSO) [44, 67, 107–109]. Studies in Macau highlighted the exclusion of MDWs from minimum wage policies [69] and the lack of a standard labor contract and contract termination subsidies [34, 110]. A study conducted in mainland China, where there is no specific visa category or immigration policy for MDWs, discussed MDWs’ barriers to accessing care and other public services as a result of their undocumented or irregular immigrant status [35]. However, three studies conducted in Hong Kong described maternity protections stipulating that MDWs in Hong Kong cannot be terminated if they become pregnant, although enforcement appeared inconsistent [71, 111–113].

Sources discussed COVID-19 policies in Hong Kong, Macau, Malaysia, Singapore, and Taiwan. This was most salient among sources focused on Hong Kong, with peer-reviewed studies and newspaper articles discussing issues including lack of arrangements of quarantine spaces for live-in migrants, high financial consequences for violating pandemic measures, exclusion from the government “Cash Payout Scheme,” which gave HKD$10,000 to all adult Hong Kong permanent residents, and COVID-19 testing and quarantine mandates solely targeting MDWs [103, 114–129]. Two peer-reviewed studies explored effects of the Singapore COVID-19 Circuit Breaker lockdown on MDWs, with targeted efforts to disperse MDW gatherings and revoke work permits [130, 131].

There was limited evidence of policies addressing post-migration factors. Only the Philippines government websites in the gray literature indicated policies and government programming to support MDWs’ repatriation, reintegration, financial management and entrepreneurship after returning permanently to the Philippines [132, 133].

The gray literature analysis revealed that various MDW sending and receiving governments have introduced policies to address pre-migration factors, primarily aiming to address the illegal and abusive recruitment practices by migration industry actors. These are discussed in the section on Migration Industry.

#### Living and working conditions

Living and working conditions was the second most salient theme across the included records (n = 193), with 119 gray literature and 76 peer-reviewed articles. In terms of working conditions, frequently identified sub-themes included surveillance and control in the workplace, restrictions on MDWs’ mobility and rest, physical and verbal abuse by employers, overwork, salaries being deducted or withheld, having contracts terminated because of MDWs’ illness or pregnancy (even in jurisdictions where MDWs have maternity protections), and poor interpersonal relationships with employers involving mistreatment and miscommunication.

A survey of 2,017 Filipino and Indonesian MDWs conducted in Hong Kong found that 61.7% worked 13 to 16 hours per day and 34.5% worked on their rest days [22]. In another study of MDWs from the Philippines, Indonesia, Thailand, and Sri Lanka working in Hong Kong, almost half reported experiences of psychological abuse, one-fourth had experienced physical abuse, and one-third reported incidents of sexual abuse in the workplace [134].

In a survey of 201 Indonesian MDWs in Singapore, one-third reported not having rest-days [135], while another study of 303 Filipino workers in Singapore found that over 20% experienced at least one of the following conditions in their current employment: delayed salary payment, no weekly rest-day, and unsafe working conditions [102]. Studies conducted in Hong Kong, Malaysia, Singapore, and Taiwan found that MDWs experienced greater workloads, more intensive work schedules and responsibilities, increased vulnerability to abuse, and salary deductions or delays in payment during the COVID-19 pandemic [44, 91, 114, 131, 136]. A Hong Kong-based NGO report from the gray literature stated that 98% of its MDW clients complained of being overworked during the pandemic [137].

Some studies described cases of positive relationships between MDWs and their employers. This included financial support extending to household expenses and healthcare needs for MDWs’ families, and reproductive care for MDWs [138–141].

Nearly all studies were conducted with live-in MDWs. Key findings relating to living conditions included insufficient food, inappropriate sleeping arrangements, lack of access to toilets, and no fans or air conditioning in hot weather. In peer-reviewed literature and NGO reports, MDWs in Malaysia and Hong Kong also described being required to be “on call” at all hours [67, 107, 142, 143].

#### Community resources

Among 120 included records describing community resources, with 69 and 51 records from the gray and peer-reviewed literature respectively, the principal theme was social support from other MDWs, with additional findings relating to family, rest-day activities and social gatherings, shared identities and cultures, and activism. MDWs described relying significantly on their host country social networks, which were primarily comprised of other MDWs from the same country of origin, for emotional, financial, and informational support.

A study in Hong Kong found that those who regularly met up with friends on days off experienced better working conditions, including fewer working hours and being less likely to be required to work on rest-days or experience salary delays and underpayment [63]. A Singapore-based study found correlations between levels of social connectedness among Filipino and Indonesian MDWs and higher general quality of life scores [31], and another study with a predominantly Filipino sample described inverse associations between stress levels and social support through mobile phones [144]. Conversely, survey results of 1,892 Filipino and Indonesian MDWs in Hong Kong found that daily communication with family back home was associated with poorer mental health status while working overseas [145].

Multi-country studies conducted in Hong Kong, Singapore, and the Philippines found that, for those preparing to migrate overseas, pre-migration contacts—the majority of whom were existing MDWs—were a key resource for information, resources, and securing employment [146, 147].

MDWs appeared to be less reliant on organizations for support and access to resources than they were on social networks and family, though some did report seeking health-related, employment-related, and legal information from NGOs and churches [103, 111, 148–151].

#### Migration industry

87 gray literature and 12 peer-reviewed articles discussed the migration industry. Identified determinants primarily concerned migration debt, the link between pre-migration exploitation and peri-migration vulnerability and abuse, and lack of support from recruitment agencies in addressing unlawful employment conditions after MDWs were placed overseas.

Studies found that practices of recruitment agencies based in MDWs’ countries of origin prior to migration significantly determined MDWs’ vulnerability and precarity throughout migration and work overseas. For example, both peer-reviewed and gray literature in Singapore described how recruitment agencies based in Myanmar, Indonesia, and Philippines imposed high fees for recruitment and training, forcing migrants into pre-migration debt (i.e., debt bondage), and frequently sided with employers over MDWs during labor or salary payment disputes [81, 103, 105, 152]. Amnesty International conducted an extensive investigation into training centers in Indonesia, revealing that the centers have deceived and mistreated prospective workers during the recruitment process—confiscating personal documents, requiring them to sleep in overcrowded facilities, completing unpaid work for the recruitment agencies, restricting mobile phone use, family visits, and external counsel, and forcing workers to receive contraceptive injections—even prior to being deployed for domestic work overseas [153]. Amnesty International demonstrated how factors in the pre-migration phase have continued influence on MDWs throughout the migration journey, linking coercive and illegal recruitment practices by Indonesian recruitment agencies to MDWs’ vulnerability and entrapment in cycles of debt, exploitation, and abusive working conditions after arriving in Hong Kong [154–157]. Hong Kong-based NGO Mission For Migrant Workers (MFMW) reported in its 2021 Service Report that one out of every three of its clients was a victim of illegal recruitment practices and overcharging [137].

Ninety percent of Indonesian respondents to a survey (n = 201) conducted in Singapore reported repaying recruitment debt accrued prior to migration over months of salary deductions after moving overseas, and only approximately 1% were able to provide itemized breakdowns of their agency fees to place them overseas [152]. A study in Malaysia highlighted the debt bondage situations of Indonesian MDWs who were forced to accept adverse or unsafe working conditions [158]. A survey of 1045 Filipino MDWs conducted by a migrant rights NGO found that, on average, 35.6% of respondents’ incomes were spent on loans and agency fees [159]. The survey also found that 96% had been charged illegal amounts (above 10% of their first month’s salary according to Hong Kong employment law) by their recruitment agencies [160].

Similarly, a study in Macau using qualitative methods also identified high recruitment fees and exploitation or deception by recruitment agencies in the pre-migration phase as significant health determinants for Filipino MDWs after moving overseas [34]. Another qualitative study found significant delays and inconsistencies in agency support for Indonesian MDWs who had become undocumented in Hong Kong and Macau [110]. Ethnographic fieldwork in Taiwan revealed Filipino agencies’ support of employers over MDWs, with an agent even encouraging an employer to illegally terminate the employment of a domestic worker who had developed an illness [140]. A qualitative study of migration between Indonesia and Malaysia discussed agencies’ roles in promoting perceptions of Indonesian MDWs as passive and subservient to employers—while this increased employability or desirability of MDWs, the researchers also emphasized the risks to MDWs that such stereotypes and expectations presented [107].

Among the included government sources from the gray literature, only the Hong Kong, Philippines, and Indonesian government websites detailed policies and resources to address the migration industry and pre-migration factors. The Hong Kong government developed an Action Plan to tackle employment and recruitment risks of MDWs, while the Philippines government released statements regarding reports of Filipino MDW abuse, exploitation, and forced labor in Hong Kong [161, 162]. The Cabinet Secretariat of Indonesia described plans to establish agreements with top MDW receiving countries to facilitate migrant placement and eliminate migrant trafficking and forced labor, addressing the pre-migration roots of migrant vulnerability and abuse [163].

#### Stigma and discrimination

46 gray literature and 17 peer-reviewed sources focused on social discrimination, stigmatization, and public perceptions of MDWs among general populations in host locations.

Studies conducted in Hong Kong and Singapore highlighted the media’s exclusion of MDW perspectives and their role in contributing to MDWs’ othering [114, 130, 164, 165]. A study that administered self-report surveys among MDWs in Macau found that levels of perceived discrimination were significantly associated with depression and anxiety [98].

Gray literature news articles in Singapore described widespread perceptions of MDWs as “second-class workers” and a “cultural threat,” and reported on MDWs’ experiences of unfair treatment in institutions such as the legal system [166–168].

#### Intrapersonal resources

Among the 43 identified articles—comprising of gray literature (n = 13) and peer-reviewed (n = 30) evidence—discussing intrapersonal resources, key determinants included positive thinking, reframing their work and living situation, self-development to increase opportunities for social mobility, religion and spirituality, and enjoying the freedom and independence offered by migration.

Some dysfunctional coping strategies were identified, such as substance use, behavioral disengagement, denial among Indonesian MDWs in Taiwan [169], and emotional numbness among workers in Macau [170].

#### Knowledge and behaviors

The primary findings from 19 gray literature and 21 peer-reviewed literature articles discussing knowledge and behaviors included MDWs’ health literacy, digital literacy, and knowledge of their employment rights and access to health services in the host country or territory. Key behaviors identified were mostly related to preventive health behaviors, such as sexual risk-taking and vaccination. For example, in a Hong Kong study of 2,012 participants, 84.9% of Filipinos and 62.6% of Indonesians had received or were willing to receive a COVID-19 vaccine at the time of data collection; higher educational attainment, higher COVID-19 knowledge scores, and seeking information from government sources rather than interpersonal networks were associated with higher COVID-19 acceptance [24].

In another survey of Filipino and Indonesian MDWs in Hong Kong, only one-third fully knew their pregnancy rights, with 30% incorrectly believing that it was legal for their employers to terminate their contract if they became pregnant [112]. One Hong Kong-based study found that, among MDWs from the Philippines, Indonesia, and Thailand who had become pregnant while working in Hong Kong, 61% of pregnancies had been “unwanted,” indicating low contraception knowledge or utilization [171]. In Hong Kong and Singapore, gray literature news articles described how MDWs’ entrapment in cycles of debt was linked to low financial knowledge and poor management of finances across the migration journey [172, 173].

A survey of 1,368 Filipino MDWs in Macau found that participants who did not live with their employers engaged in riskier sexual behaviors and had higher rates of gambling than live-in participants [93, 174]. Only one peer-reviewed article on MDW sexual health testing was identified in the review [95]. It reported that fewer than 25% of the 1,363 Filipino MDWs in the study had been previously tested for HIV, with a zero prevalence of HIV and syphilis based on rapid testing results. Another study conducted in Macau reported that only 37% of Filipino MDW participants had “sufficient” general health literacy scores, with English proficiency and being over the age of 30 associated with higher health literacy [36].

#### Healthcare access

A total of 33 sources from the peer-reviewed (n = 14) and gray literature (n = 19) focused on working conditions, limits on healthcare coverage, and policy restrictions as barriers to accessing formal healthcare services. Studies in Hong Kong and Singapore described access to care as largely determined by employers’ willingness to allow their workers to seek care [76, 151]. A mixed-methods study of 30 MDWs in Hong Kong found that healthcare access was associated with MDWs’ technology and internet access, especially via up-to-date health information and telemedicine appointments during the COVID-19 pandemic, and support from employers and friends [175].

A study of MDWs in Singapore who had worked overseas long-term found that while employers were legally required to purchase health insurance coverage for their employees, most chose the most basic health insurance [75]. Some insurance companies even refused to renew the plans of older MDWs or MDWs with chronic illnesses [75]. A sample of Filipino MDWs in Macau reported only having mandatory accident insurance coverage, with most not having access to services like preventive care and sexual and reproductive services [34]. In a study of Indonesian MDWs in Hong Kong, few accessed dental care because only dental emergencies were covered by their health insurance [33].

Evidence from mainland China highlighted the lack of MDW working visas as a healthcare access barrier [35]. Similarly, undocumented migrants in Malaysia described their fears of being reported to the Immigration Department if they utilized public healthcare services [141].

Filipino women in Macau experienced challenges accessing HIV-testing services, which were predominantly provided by private clinics that were closed on Sundays (most MDWs’ rest-days) and required additional appointments and fees [93]. Some overcame these barriers by seeking testing services from NGOs instead.

Some studies discussed discrimination within healthcare settings and distrust of providers as a barrier to health care. MDW participants in Singapore described care providers’ negative judgments and seeming unwillingness to treat MDWs [76]. Filipino MDWs in a Macau-based study referenced language barriers and a lack of trust [34].

### Interventions

There was a total of six studies describing interventions to address MDW social determinants of health. They were conducted in Singapore (n = 4), Hong Kong (n = 1), and Macau (n = 1). Four studies were interventional and two were observational in design. The interventions primarily targeted MDW knowledge and behaviors. The characteristics and details of the studies are displayed in Table 6.

**Table 6:** Characteristics and findings of included studies on interventions addressing MDW social determinants of health.

Author(s)	Study Location	Study Design	Target Population [N]	Content of Intervention	Identified Health Determinants	Primary Outcome [Assessment Method]	Main Findings
Barua, R et al., 2020 [176]	Singapore	Interventional, quantitative, RCT	Female MDWs [243]	Peer-support groups meeting monthly for 3 hours over 9 months; standardized curriculum on savings, budgeting, remittances, and financial goals and business plans	Knowledge and behaviors	Whether participants reported savings and reported amount of savings [survey]	High attrition, low intervention take-up by invited participants; assignment to treatment had negative effects on whether participants reported savings and amount of savings reported
Eng et al., 2021 [73]	Singapore	Observational, quantitative, retrospective review	MDWs [322]	Information and counselling on cervical cancer screening and tests scheduled for consenting participants; counselling for cytology triage and appointment for colposcopy for positive tests	Knowledge and behaviors, Healthcare Access	Appointment attendance and rates of follow-up for positive tests [retrospective review of medical records]	68.6% attended scheduled appointments; 51% who had positive tests declined cytology triage or colposcopy
Liem et al., 2022 [177]	Macau	Observational, mixed methods convergent	Program implementors and stakeholders [8] Overseas Filipino workers [25]	Kumusta Kabayan mobile application program—Filipino version of WHO Step-by-Step online self-help intervention for people with depressive symptoms	Knowledge and behaviors; Healthcare Access	Implementor and stakeholder perspectives on implementation, feasibility, and success; user experiences [survey, in-depth interview]	Implementors and stakeholders had positive perceptions of implementation, feasibility, and effectiveness; concern about infrastructure and sustainability Some misconception about program content; participants who completed used almost daily; barriers included time, work, and tiredness
Shrestha and Yang, 2019 [102]	Singapore	Interventional, quantitative, RCT	Filipino MDWs [303]	Provision of info on labor laws and resources regarding changing employers in Singapore	Knowledge and behaviors	Participants’ knowledge about legal rights and attitudes towards changing employers [survey]	Significant improvement in legal knowledge; some immediate change of employers; increased intentions to change employers and improve work conditions; >70% reported sharing info with friends
Wong et al., 2019 [178]	Singapore	Interventional, quantitative, RCT	Filipino MDWs [39]	4 weekly 3-hour group trainings administered by 2 clinical psychology trainees in English; training adapted from CBT-based paraprofessional manuals to train refugees to develop skills to address depression	Knowledge and behaviors	Knowledge about depression and CBT, professional help-seeking for depression [survey]	High attendance and program satisfaction; no significant difference in knowledge or professional help-seeking between treatment and control
Zhou et al., 2020 [179]	Hong Kong	Interventional, quantitative, quasi-experimental	MDWs [191]	Workshops on personal finances, investment, banking, retirement and insurance, investment scams, entrepreneurship, communication and balancing needs	Knowledge and behaviors	Financial knowledge and behaviors, general and financial self-efficacy [survey]	Treatment group had improved knowledge and awareness of financial risks and good management practices, increased self-efficacy, long-term attitude and behavioral changes and increased engagement on financial matters with family members back home

Across the six studies, four of the studies targeted only Filipinos, while two studies also included a minority of Indonesian participants. There were generally mixed findings regarding the efficacy of the six interventions. No evidence for efficacy was identified for two randomized controlled trials (RCTs) conducted in Singapore related to financial literacy and mental health paraprofessional training [176, 178]. However, another RCT of an informational intervention in Singapore found increases in participants’ knowledge, attitudes, and behaviors regarding employment rights and changing employers [102]. A quasi-experimental study of a financial literacy and skills program in Hong Kong was found to increase knowledge and self-efficacy of participants compared to the control group [179]. An observational study of a digital mental health intervention culturally adapted for Filipino migrants in Macau identified generally positive experiences regarding implementation, feasibility, and effectiveness among program implementations, researchers, and users, but no evidence of program effectiveness was reported [180].

## Discussion

The current scoping review synthesized findings from 173 peer-reviewed sources and 276 gray literature sources on MDWs health and wellbeing, social determinants, and interventions to improve their health and address associated determinants. Most of the studies (130/173, 75.1%) used qualitative methods. Only six studies of interventions were identified, mostly focusing on income-related factors, and with generally poor or untested efficacy.

Most of the included records were based in Hong Kong and Singapore and focused on migrants from the Philippines and Indonesia. While this affects the generalizability of the findings to all MDWs in the Western Pacific, these patterns are reflective of the geographical distributions of MDWs—Hong Kong and Singapore host the largest proportions of MDWs, while the Philippines and Indonesia are the largest sending countries of MDWs working in the region (Table 1).

There were mixed findings in terms of physical health, mental health, and well-being outcomes among MDWs. While surveys in Macau and Singapore found generally positive self-reported health [31, 93], a survey conducted in Hong Kong found that participants had worse self-reported health than the general population [22]. Salient themes included chronic conditions, work-related pain and injury, and mental health problems (particularly anxiety and depression), which is generally aligned with findings of women migrants and migrant workers globally [180–183]. The studies illustrated disparities in health outcomes between MDW sub-populations. For example, two studies conducted in Singapore concluded that MDWs from Myanmar were at higher risk for poor physical and mental health outcomes than other MDW participants [31, 77]. While all the studies focused on the health outcomes of MDWs during the peri-migration phase, there was limited discussion of the long-term health outcomes among MDWs who had been working overseas for significant periods of time [74, 75].

The scoping review identified no studies on health outcomes of MDWs in Malaysia and Taiwan, constituting a significant literature gap as both are key destinations for migrant domestic workers. With few exceptions [93, 95, 141], included studies also lacked exploration of gendered health issues, such as sexual and reproductive health outcomes, which the WHO identified as a key outcome among women migrant laborers around the world [181].

Review findings are broadly aligned with the IOM framework on migration and the social determinants of health, with several key exceptions. Evidence included in the current review considerably expanded the theme of “lifestyle factors” to include intrapersonal resources (e.g., coping strategies), knowledge, and behaviors. We also expanded the framework to capture the nuanced situation of live-in workers, whose living and working conditions are inextricably linked. Finally, although the IOM framework portrays social factors as distal health determinants, the analysis found greater salience of proximal strengths-based factors, such as local social networks for emotional, informational, financial, and other supports, compared to distal factors like stigma and discrimination.

The most salient health determinants theme among the included peer-reviewed and gray literature articles was policies and governance. While studies on policies and governance were conducted across all key MDW destinations in the region, only one was conducted across multiple host locations. This limited the ability for inter-jurisdiction comparison. However, there were consistent themes across the peer-reviewed and gray literature articles, with the most notable including live-in requirements, maternity protections, insufficient enforcement of MDW protections, COVID-19 policies, and exclusion of MDWs from local labor laws. Among the key MDW destinations in this study, only Hong Kong and Taiwan have introduced a standard minimum wage for MDWs, though both are lower than the minimum wages for the general population. In 2022, the Taiwanese government increased the monthly MDW minimum wage by 17.6%, to NT$20,000 (US$618^a^), while in 2023, it was increased by 3%, to HKD$4,870 (USD$623 According to currency conversion rates in October 2023) per month in Hong Kong [184]. However, activists and unions contend that the Hong Kong minimum wage remains insufficient to meet daily needs and keep up with inflation, arguing for an increase to HKD$6,100 (USD$784). In Taiwan, the increase only affected newly arriving migrants and did not apply to MDWs with existing contracts.

Moreover, the scoping review revealed that, even with the existence of minimum wage and maternity protections in Hong Kong, gaps remain in enforcement, resulting in comparable outcomes for MDWs in terms of working conditions and rights violations across study locations. Findings were in alignment with policy contexts of MDWs outside of the Western Pacific region, pointing to global gaps in legal recognition of domestic workers’ rights and well-being, and disparities in migration policy between domestic and non-domestic workers [185–187].

In the top destinations in the Western Pacific, migrants who enter on MDW visas and work permits do not have pathways to attaining permanent residency status, with exceptions granted only in some cases of marriage to local citizens [188–192]. While in 2022, the Taiwanese government announced plans for migrant workers classified as “intermediate-skilled” workers to become eligible for permanent residency, it is unclear whether this designation will apply to domestic work [193].

Similar to previous scoping reviews on MDW health, living and working conditions was a prominent theme. The peer-reviewed and gray literature generally illustrated negative interpersonal relationships between MDWs and their employers. Particularly for live-in migrants, employers largely shaped outcomes both within and outside of the workplace, dictating access to rest, privacy, social networks, and basic services like healthcare.

The gray literature evidenced how widespread reports and high-profile cases of MDW abuse and exploitation in workplaces have even led to direct intervention from sending countries. Between 2014 and 2019, the government of Myanmar introduced a ban on migration for domestic work to Singapore, in response to cases of MDW abuse, debt bondage, and trafficking [115]. Similarly, from 2009 to 2011, the Indonesian government imposed a ban on labor migration to Malaysia due to reports of migrant worker abuse [194].

Only one study, conducted in Macau, described the living conditions of live-out workers, with the majority of participants describing inadequate living and sleeping conditions in crowded boarding houses [34]. This limited the ability to analyze the ways in which living conditions directly shape health outcomes for MDWs [34]. The Singapore Household Services Scheme (HSS)—through which approved companies employ migrant workers to provide ad-hoc domestic services to households, as an alternative to a live-in full-time MDW—was not discussed in the studies, but may be an interesting case study for live-in/live-out comparison, which can be conducted in Macau [195].

Though studies focused on the employer-employee relationship, few incorporated employers’ perspectives. One qualitative study interviewed MDW employers in Macau, with participants describing concerns about their Filipino employees’ competence, particularly relating to childcare, suspicions of stealing, and negative judgments about their cleanliness and morals [170]. The ILO underscored the importance of engaging employers of MDWs, particularly in balancing MDW health and well-being with employers’ financial priorities, need for workers, and right to privacy in the workplace-household [196, 197].

Consistent with social capital theories and the stress-buffering hypothesis [198–200], findings suggest that community resources promote migrant health and well-being and provide protection from adversities. However, one study conducted in Macau highlighted some Filipinos’ experiences of distrust and fighting within social networks, possibly substantiating findings of unexpected positive associations between severity of mental health symptoms and social network support among a Filipino sample in another study [34, 201]. Another study in Hong Kong found that daily communication with family back home was linked to poorer mental health [145]. These findings warrant further exploration of the specificities of MDW social capital—such as intersectional factors like gender and social class—and their linkages to health.

There were significant gaps in the literature on the migration employment industry and access to healthcare, which were among the least-discussed themes in the included literature. Studies found that the recruitment industry created situations of bonded labor and adverse working conditions for MDWs, aligning with the IOM and Zimmerman frameworks describing pre-migration vulnerability [53, 56]. However, the studies provided little data on the role of host governments in organizing and regulating migration. Though migration intermediaries are primarily located in sending countries, experts highlight the need for cross-border partnership between sending and host governments and transnational legal frameworks to prevent modern slavery [202, 203].

The studies indicated that the underlying determinant of healthcare access was deficiencies in policy. Consistent with data on women migrants globally, articles discussed how immigration policies and restrictive work visas provided limited health coverage and affected migrants’ ability to obtain health insurance [181, 204–206]. A recent intervention model developed by the ILO highlighted the need for receiving governments to increase and regularize social protections to tackle vulnerability and increase healthcare access for migrant workers employed in the informal economy [207].

The findings of MDWs’ perceived discrimination, distrust, and language barriers in healthcare settings were also supported by global data on barriers to health for migrants [208, 209]. However, given the finding that access to care for MDWs was mostly determined by employers, a prominent gap in the literature was an exploration of employers’ decision-making regarding their workers’ healthcare needs and coverage [76, 141].

The analysis identified the COVID-19 pandemic as a cross-cutting theme. The findings of restrictive and inequitable policies and worsened working and living conditions aligned with the broader literature that migrant laborers around the world were subjected to increased victimization and exploitation during COVID-19 [210]. However, given the live-in nature of domestic work, employers’ perspectives regarding their health priorities and fear of COVID-19 infection, as well as post-COVID-19 policy and sociocultural shifts, need further attention.

There was limited discussion among the studies on factors related to the migration industry during the pandemic. This is a notable gap given that the Indonesian government introduced a temporary halt on migration to Malaysia in light of allegations of exploitation and trafficking of Indonesian migrants in 2022 [211].

The ILO and Zimmerman et al.’s frameworks on vulnerability throughout various migration phases underscore the importance of viewing migration through a temporal lens. The analysis contributes to the literature by evidencing how pre-migration factors, particularly the migration industry, can influence MDW outcomes and MDWs’ vulnerability throughout the migration journey. Most of the research on MDWs has centered on the peri-migration phase, particularly living and working conditions. Discussion of post-migration health determinants and interventions was identified only in the Philippines government websites from the gray literature [132, 133]. Additionally, the reliance on cross-sectional data hindered the ability to draw conclusions on the health implications of accumulated stressors and exposures throughout MDWs’ cyclical and serial migration trajectories and for MDWs who had worked overseas for significant periods of time.

This scoping review identified limited interventional studies addressing MDW health outcomes and social determinants of health. There were no studies that found interventions in Malaysia and Taiwan. Despite homogeneity in findings across study locations regarding occupational injury and chronic health conditions, no interventions focused on physical health outcomes. Moreover, the interventions addressed proximal health determinants such as financial literacy and mental health self-help. This is supported by findings from a systematic review of interventions on migrant worker health, which identified limited structural-level interventions, with the majority intervening on proximal health determinants [212]. Taken together, these findings indicate a need for further research on interventions for migrant and MDW health, particularly in addressing the structural determinants of health.

The high proportion of studies using qualitative methods affected generalizability of the study findings to broader MDW populations, especially given relatively small study sample sizes, and with few studies based on random sampling or probability sampling approaches including respondent driven sampling. Besides studies that conducted ethnographic fieldwork, no longitudinal studies were identified. This limited our ability to understand causal relationships between health determinants and health outcomes, as well as to explore accumulated exposures through repeated migration. There was limited evidence of instrument validation in the studies. A substantial proportion of studies administered interviews and survey questionnaires in English only, and with scales that had not been validated in the study population. Among the key sending countries, only the Philippines has English as a national language, but there are wide ranges of proficiency in English language literacy. Studies primarily used convenience sampling, conducted in public spaces on the weekend, when MDWs are typically on their weekly rest-day. However, given these findings indicate access to rest-days is not guaranteed for all MDWs, study samples may potentially be skewed towards MDWs with relatively favorable living and working conditions.

## Recommendations

As much of the literature was focused on MDW health outcomes and determinants in Hong Kong and Singapore, future peer-reviewed and grey literature should explore under-studied MDW destinations such as Malaysia and Taiwan. More data is needed on under-represented migrant populations from Myanmar, Thailand, Vietnam, and countries in South Asia including India, Nepal, and Bangladesh, who may face greater barriers to health and well-being. Cross-population comparisons would contribute to understandings of sub-group vulnerabilities and inform tailored intervention.

Future research should incorporate quantitative and mixed methods, to allow for quantification and comparison of health outcomes and determinants. Studies with designs that incorporate causal inference are needed to estimate the effects of determinants on health and the impact of accumulated exposures throughout the migration journey. Validation of study instruments among MDW populations is also critically needed [213–217]. Studies should consider data biases relating to instrument translations and the choice to use unvalidated measurements in a language that may not be well understood by the study population. Innovations in sampling methods are needed, particularly given the private setting of domestic work. For example, respondent driven sampling approaches or venue-based sampling may yield different results from sampling methods that rely on employer referral through household telephone surveys. Additional work is needed to understand the effect of social networks and forms of social capital that may improve and protect the health and wellbeing within this population. Future research should also include a critical appraisal of studies on MDW health determinants and interventions.

From a broader policy perspective, host governments should take substantial responsibility to ensure the health and welfare of migrant domestic workers. One possible approach would be to enroll MDWs in health registries, as is the case in Denmark, so their health and service utilization can be monitored [218, 219]. A registry system can also ensure they are being provided the necessities promised in their contracts and supported by labor laws including proper housing, wages, and access to health care. NGOs play a vital role in reaching MDWs and policies should enable them to employ staff from MDWs’ home countries, to provide key cultural and linguistic linkages for these populations. Additional work is needed to center the voices and lived experiences of MDWs in solidarity to advocate for health policy that creates an optimal context for health and ensures equitable health outcomes.

Future studies should explore the causal relationships between determinants and health outcomes, particularly through cross-country comparisons with common metrics. Structural health determinants such as healthcare access and the migration industry also require further study. To gain a complete understanding of MDW health barriers, researchers should also engage key stakeholders including MDW employers and recruitment agencies.

The actions taken by host governments, governments in sending countries, and NGOs to maintain the health and welfare for migrant domestic workers vary considerably by context. Additional cross-sectoral and inter-governmental efforts are needed to harmonize this agenda. Multi-sectoral partnerships, including with MDWs advocacy groups and academic partners, are needed to set strategic priorities and co-design interventions, including innovative digital health applications that can be adapted for use in multiple settings, contexts, and sub-populations [220]. Future studies can explore multi-level interventions, considering both proximal and structural factors of MDW health and well-being.

## Limitations

This scoping review had several limitations. The review only included literature published in English, which is not an official language in most of the Western Pacific countries under study. The study only included peer-reviewed literature conducted in host locations, limiting understanding of health determinants pre- and return-migration. As the scoping review did not assess the quality of the included studies, the authors were unable to draw conclusions on the overall strength of the available data. The IOM and Zimmerman frameworks used in this study may not have been the most suitable for capturing the situation of MDWs, since they were not developed specifically for MDWs or migrants in the Western Pacific region.

## Conclusion

This scoping review is the first to conduct a comprehensive analysis of the available peer-reviewed and gray literature on migrant domestic workers’ health and well-being outcomes, health determinants, and related interventions in the Western Pacific region. The findings indicate a mix of positive and negative physical health, mental health, and well-being outcomes among MDWs, with some sub-population differences. Key health determinants included intrapersonal resources, knowledge, and behaviors, living and working conditions, community resources, stigma and discrimination, healthcare access, the migration industry, and policies and governance. The findings generally described negative effects of the identified determinants on MDW health and well-being. Limited literature was found on interventions relating to MDW health outcomes and determinants. Future research should incorporate perspectives of employers and migration industry actors and focus on MDWs in territories outside of Hong Kong and Singapore. Studies should incorporate more quantitative methods, and longitudinal study designs will allow for the establishment of causality between health determinants and outcomes. More interventional studies are required to establish the effectiveness and implementation of interventions relating to MDW health and well-being.

## Supporting information

S1 Checklist Preferred Reporting Items for Systematic reviews and Meta-Analyses extension for Scoping Reviews (PRISMA-ScR) checklist. (DOCX)

S1 Data Complete peer-reviewed and gray literature sources. (XLSX)

S1 Appendix Peer-reviewed literature search strategy for PubMed. (DOCX)

S2 Appendix Peer-reviewed literature data extraction form. (DOCX)

S3 Appendix Grey literature sources and search strategy. (DOCX)
