# Supplementary material for: The social determinants of migrant domestic worker (MDW) health and well-being in the Western Pacific Region: A Scoping Review
Source: PLOS Glob Public Health. 2024 Mar 27;4(3):e0002628. doi: 10.1371/journal.pgph.0002628 (PMC10971684; doi:10.1371/journal.pgph.0002628)
Supplement: S1 Appendix — (DOCX) [file pgph.0002628.s003.docx]

Appendix 1. Peer-Reviewed Literature Search Strategy for PubMed

| **Search Block** | **Keywords (titles, abstracts, subject headings)** |
| --- | --- |
| 1. MD | ("transients and migrants" [MeSH] OR "domestic work*"[tw] OR "migrant work*"[tw] OR "foreign work*" [tw] OR "domestic labor"[tw] OR "migrant labor"[tw] OR "labor migra*"[tw] OR "domestic help*"[tw] OR helper[tw] OR "overseas work*"[tw] OR "overseas Filipino work*"[tw] OR "migrant care work*"[tw] OR "live-in care"[tw] OR (foreign[tw] AND maid*[tw]) OR "home care work*"[tw] OR "household service work*"[tw] OR "transnational work*"[tw] OR (transnational[tw] AND "domestic work*"[tw]) OR (transnational[tw] AND "care work*"[tw]) OR OFW[tw] OR FDW[tw] OR MDW[tw]) |
| 1. Region | ("Polynesia"[Mesh] OR "Melanesia"[Mesh] OR Australia[Mesh] OR Brunei[Mesh] OR Cambodia[Mesh] OR China[Mesh] OR Fiji[Mesh] OR Guam[Mesh] OR "Hong Kong"[Mesh] OR Japan[Mesh] OR Kiribati[Mesh] OR Laos[Mesh] OR Macao[Mesh] OR Macau[Mesh] OR Micronesia[Mesh] OR Mongolia[Mesh] OR Nauru[Mesh] OR "New Caledonia"[Mesh] OR "New Zealand"[Mesh] OR Niue[Mesh] OR Palau[Mesh] OR "Papua New Guinea"[Mesh] OR Philippines[MeSH] OR "Pitcairn Island"[Mesh] OR "Republic of Korea" [Mesh] OR Samoa[Mesh] OR Singapore[Mesh] OR Tokelau[Mesh] OR Tonga[Mesh] OR Tuvalu[Mesh] OR Vanuatu[Mesh] OR Vietnam[Mesh] OR "australia"[tw] OR "brunei"[tw] OR "cambodia"[tw] OR "china"[tw] OR "Cook Islands"[tw] OR "fiji"[tw] OR "French Polynesia"[tw] OR "guam"[tw] OR "Hong Kong"[tw] OR "japan"[tw] OR "micronesia"[tw] OR "laos"[tw] OR "macau"[tw] OR "macau"[tw] OR "Marshall Islands"[tw] OR "micronesia"[tw] OR "mongolia"[tw] OR "micronesia"[tw] OR "New Caledonia"[tw] OR "New Zealand"[tw] OR "polynesia"[tw] OR "palau"[tw] OR "Papua New Guinea"[tw] OR "philippines"[tw] OR "Pitcairn Island"[tw] OR "Solomon Islands"[tw] OR "korea"[tw] OR "samoa"[tw] OR "singapore"[tw] OR "polynesia"[tw] OR "tonga"[tw] OR "micronesia"[tw] OR "vanuatu"[tw] OR "vietnam"[tw] OR "Viet Nam"[tw] OR "Wallis and Futuna"[tw]) |
| Final Search | 1 AND 2 |
