# Supplementary material for: The social determinants of migrant domestic worker (MDW) health and well-being in the Western Pacific Region: A Scoping Review
Source: PLOS Glob Public Health. 2024 Mar 27;4(3):e0002628. doi: 10.1371/journal.pgph.0002628 (PMC10971684; doi:10.1371/journal.pgph.0002628)
Supplement: S2 Appendix — (DOCX) [file pgph.0002628.s004.docx]

2. Peer-Reviewed Literature Data Extraction Form

| **Section** | **Data Item** | **Description or Response Options** |
| --- | --- | --- |
| General Information | Title | Title of Publication |
|  | Contact Information | Email |
|  | Authors | Names of all authors |
|  | Year | Year of Publication |
|  | Country | Country or territory of study |
|  | Source | Journal name |
| Study Information | Study Type | Observational or Interventional |
|  | Study Design | E.g., Cross-sectional, longitudinal, RCT |
|  | Data Type | Quantitative, Qualitative, or Mixed-Methods |
|  | Sampling Method | E.g., convenience, random, cluster |
|  | Study Length | In years or months |
|  | Outcome Type | Health outcomes, health determinants, or intervention |
|  | Instruments Used | E.g., interviews, survey |
|  | Control or Comparison Group | Yes/No |
|  | If Yes, Control or Comparison Group Description | Description, if control/comparison used |
| Study Setting and Population | Total Population Reported | # |
|  | Total Population Analyzed | # |
|  | Population Description | e.g., demographic information |
| Health Outcomes | Types of Health Outcomes | Physical health or mental health and well-being |
|  | Description of Health Outcomes | Details of outcomes |
| Health Determinants | Health Determinants Theme | Intrapersonal resources, knowledge and behaviors, living and working conditions, community resources, stigma and discrimination, healthcare access, migration industry, policies and governance |
|  | Description of Health Determinants | Details of health determinants |
|  | Health Determinants Effects or Outcomes | Description of correlates of determinants detailed above |
| Interventions | Type of Intervention | E.g., informational, clinical |
|  | Organization that Delivered the Intervention | E.g., researchers, NGO |
|  | Objective(s) | Stated objectives |
|  | Key Activities | Details of intervention |
|  | Key Actors | Any other stakeholders besides implementors |
|  | Implementation Details | E.g., fidelity, attrition |
|  | Effects or Outcomes | E.g., health outcomes |
| Additional Information | Funding | Funder of study |
|  | Conflicts of Interest | Any stated competing interests of authors |
|  | Comments | E.g., quotes |
|  | Related References | References from source to be screened for inclusion |
