# Supplementary material for: The social determinants of migrant domestic worker (MDW) health and well-being in the Western Pacific Region: A Scoping Review
Source: PLOS Glob Public Health. 2024 Mar 27;4(3):e0002628. doi: 10.1371/journal.pgph.0002628 (PMC10971684; doi:10.1371/journal.pgph.0002628)
Supplement: S3 Appendix — (DOCX) [file pgph.0002628.s005.docx]

3. Grey literature Sources and Search Strategy

| **Type** | **Sources** | **Search Strategy** |
| --- | --- | --- |
| Databases | Migration Policy Institute  <https://www.migrationpolicy.org/> | Search “domestic worker” in “All MPI”, apply region filter by region “Asia and the Pacific” |
|  | Open Knowledge Repository by the World Bank Group  <https://openknowledge.worldbank.org/> | Advanced Search filters:  Keyword Contains “domestic work”;  World Bank Regions Contains “East Asia & Pacific” |
|  | Reliefweb  <https://reliefweb.int/> | (“domestic work” OR “domestic worker”) AND (international OR transnational OR migrant OR overseas OR foreign) |
|  | OECD iLibrary  <https://www.oecd-ilibrary.org/> | Advanced Search filters:  All Fields Contains “domestic work” OR “domestic worker”;  AND All Fields Contains “international OR transnational OR migrant OR overseas OR foreign” |
| Government Website | **Indonesia**  Cabinet Secretariat of the Republic of Indonesia  <https://setkab.go.id/en> | “domestic worker” |
|  | **Philippines**  Department of Labor and Employment <https://www.dole.gov.ph/> | OFW, “domestic work,” “domestic worker”, helper |
|  | Department of Migrant Workers <https://dmw.gov.ph/> | Review entire website |
|  | Overseas Workers Welfare Administration  <https://owwa.gov.ph/> | News  Programs and Services  Search: OFW |
|  | Philippine Statistics Authority  <https://psa.gov.ph/> | Statistics > Social > Labor and Employment  Search: OFW |
|  | **Brunei**  Department of Labour, Ministry of Home Affairs of Brunei |  |
|  | **Hong Kong SAR**  Hong Kong Labour Department |  |
|  | Hong Kong Government |  |
|  | Immigration Department of Hong Kong |  |
|  | **Macau SAR**  Macau SAR Government Portal |  |
|  | **Singapore**  Philippine Overseas Labor Office (POLO) Singapore |  |
|  | Singapore Ministry of Manpower |  |
|  | **Taiwan**  Ministry of Labor Taiwan |  |
| Intergovernmental organizations, international NGOs, and UN agencies | International Labour Organization  <https://www.ilo.org/> | Topics > Domestic workers  Search “domestic work”, “domestic worker”  Filter search with Region “Asia” |
|  | International Domestic Workers Federation (Asia Pacific)  <https://idwfed.org/en/affiliates/asia-pacific> | Resources > filter Topic “Migrant Domestic Workers” |
|  | Migration Data Portal <https://www.migrationdataportal.org/> |  |
|  | International Organization of Migration  <https://www.iom.int/> | Search: domestic work, domestic worker  Filter Region Country: “Asia (1043)  Review websites for individual receiving and sending countries |
|  | UN Women Asia and the Pacific  <https://asiapacific.unwomen.org/en> | Search: “domestic work”, “domestic worker”  Filter Region: “Asia and the Pacific” |
|  | United Nations Population Fund  [https://www.unfpa.org](https://www.unfpa.org/) | “domestic work”, “domestic worker” |
|  | International Trade Union Confederation - Asia Pacific  <https://www.ituc-ap.org/> | “domestic work” |
|  | Global Labor Organization  <https://glabor.org/> | “domestic work”, “domestic worker” |
|  | Amnesty International <https://www.amnesty.org/en/> | Search “domestic work”  Filter Countries: “Asia and the Pacific” |
| Local Associations, NGOs, and Advocacy Groups | Asian Migrant Centre <https://www.asianmigrantcentre.org/> | Review entire website |
|  | Mekong Migration Network  <http://mekongmigration.org/> | “domestic work” |
|  | Migrante International [https://migranteinternational.org](https://migranteinternational.org/)/ | “domestic work” |
|  | Christian Action  <https://www.christian-action.org.hk/en/hong-kong/humanitarian-social-services/migrant-domestic-workers> | Review entire website |
|  | Mission for Migrant Workers  <https://www.migrants.net/> | Publications > Reports, Researches, Newsletters |
|  | HELP for Domestic Workers  <https://helpfordomesticworkers.org/en/home/> | Review entire website |
|  | HK Helpers Campaign  <http://hkhelperscampaign.com/en/> | Review “Our 3 Campaign Points” and “Resources”  *organization closed in 2016, but Facebook page still active |
| Newspaper Article | ASEAN Today <https://www.aseantoday.com/> | "domestic work”, OFW, helpers |
|  | Southeast Asia Globe <https://southeastasiaglobe.com/> | domestic work  excluded |
|  | Hong Kong Free Press <https://hongkongfp.com/> | “domestic work” |
|  | South China Morning Post | “domestic work” |
|  | The Guardian <https://www.theguardian.com/international> | “domestic workers” AND (individual searches of sending and receiving countries)  excluded |
|  | The Straits Times | “domestic work” |
|  | New York Times  <https://www.nytimes.com/> | “migrant domestic workers” |
| Pre-print databases | medRxiv <https://www.medrxiv.org/search> | "domestic work”  excluded |
|  | PsyArXiv [https://psyarxiv.com](https://psyarxiv.com/) | “domestic workers”  excluded |
| Social media | **Grassroots & Non-governmental Organizations** Filipino Migrant Domestic Worker's Union <https://www.facebook.com/FMDWU/> |  |
|  | Migrante International <https://www.facebook.com/migranteinternational> |  |
|  | HELP for Domestic Workers <https://www.facebook.com/HELPForDomesticWorkers> |  |
|  | HK Helpers Campaign <https://www.facebook.com/helpershk/> |  |
|  | PathFinders Hong Kong <https://www.facebook.com/pathfindershk> |  |
|  | Mission for Migrant Workers <https://www.facebook.com/MFMWHK> |  |
|  | International Migrants Alliance <https://www.facebook.com/intlmigrants/> |  |
|  | Centre for Domestic Employees <https://www.facebook.com/cde.singapore/> |  |
|  | Uplift your life -Community&Free Education for Foreign Domestic Workers <https://www.facebook.com/groups/migrantdomesticworkerscommunity> |  |
|  | Domestic Workers Corner Hong Kong |  |
|  | Philippine Consulate General in Hong Kong |  |
|  | Philippine Consulate General in Macau SAR |  |
|  | Foreign Domestic Worker Association for Social Support and Training - FAST |  |
|  | Hong Kong Federation of Asian Domestic Workers Unions FADWU |  |
|  | OWWA Overseas Workers Welfare Administration |  |
|  | Department of Migrant Workers (Phi)  <https://www.facebook.com/dmw.gov.ph> |  |
| Policy | **Macau**  Government of the Macao SAR Labour Affairs Bureau  <https://www.dsal.gov.mo/en/standard/nrworker_law.html> | Review “Employment for Non-resident Workers” under the “Functions” tab |
|  | **HK**  Labour Department – Foreign Domestic Helpers  <https://www.fdh.labour.gov.hk/en/home.html> | Review entire website |
|  | Immigration Department of the Government of the Hong Kong SAR  <https://www.immd.gov.hk/eng/index.html> | Review “Foreign Domestic Helpers” |
|  | **Malaysia**  Guidelines and Tips for Employers of Foreign Domestic Helpers  <https://apmigration.ilo.org/resources/guidelines-and-tips-for-employers-of-foreign-domestic-helpers> |  |
|  | **Singapore**  Ministry of Manpower-COVID advisories  <https://www.mom.gov.sg/covid-19/topics> | Review “Managing migrant domestic workers” |
|  | Singapore Statutes Online (EMPLOYMENT OF FOREIGN MANPOWER ACT 1990)  <https://sso.agc.gov.sg/Act/EFMA1990> | Review entire page |
